# Editorial: The role of exosomes and organokines in metabolic and endocrine disease

**DOI:** 10.3389/fendo.2023.1198791

**Published:** 2023-04-21

**Authors:** Qi-Yu Wang, Zhe-Zhen Liao, Jinping Lai, Xin-Hua Xiao

**Affiliations:** ^1^ The First Affiliated Hospital, Department of Metabolism and Endocrinology, Hengyang Medical School, University of South China, Hengyang, Hunan, China; ^2^ The First Affiliated Hospital, Department of Clinical Laboratory Medicine, Institution of Microbiology and Infectious Diseases, Hengyang Medical School, University of South China, Hengyang, Hunan, China; ^3^ Department of Pathology and Laboratory Medicine, Kaiser Permanente Sacramento Medical Center, Sacramento, CA, United States

**Keywords:** organokines, obesity, diabetes, metabolic syndrome, endocrine diseases, exosomes

The field of exosomes and organokines biology and metabolism is rapidly expanding. Increasing numbers of exosomal miRNA have also been used as biomarkers for metabolic diseases in clinical settings, such as miR-34a,miR-122,miR-192,miR-142, and so on (1–4). Several biological factors including miR-130a-3p and miR-3075 are packaged into exosomes and then transported into specific organs to regulate local metabolism (5–7). Novel-identified organokines (adipokines, myokines, hepatokines) have been reported to play crucial roles in the regulation of whole-body metabolism homeostasis (8–10) affecting the regulation of glucose metabolism, lipid metabolism, insulin sensitivity, oxidative stress, low-grade inflammation and so on. The goal of this Research Topic “*The Role of Exosomes and Organokines in Metabolic and Endocrine Disease*” is to highlight a wide range of exosomes and organokines originating from the metabolic organs that mediate inter-organ crosstalk and participate in the development of metabolic diseases. Six high-quality papers (Pang et al., Chen et al., Sun et al., Mei et al., Wang et al., 11) were accepted by the Frontiers editorial team for publication in the Frontiers in Endocrinology. They provide new information to the advancement of organokines and exosome-based diagnostics and therapeutic approaches for metabolic and endocrine diseases.

In this Research Topic, Pang et al. first described the expression profile of plasma exosomal long non-coding RNAs (lncRNAs) in patients with type 1 diabetes mellitus (T1DM). That study demonstrated that 162 aberrantly expressed exosomal lncRNAs including 77 up-regulated and 85 down-regulated were associated with the pathogenesis of T1DM. Kyoto Encyclopedia of Genes and Genomes (KEGG) pathway analysis was performed and indicated that these differentially expressed lncRNAs were involved in oxidative phosphorylation. This work provided a novel insight into the pathogenesis of T1DM and a foundation for using exosomal lncRNAs as potential diagnostic biomarkers and therapeutic targets for T1DM.

In the field of diabetic vascular complications (DVC), Chen et al. summarized the most recently published research suggesting the changes in the production process of exosomes in the diabetic microenvironment and the early warning role of exosomes in DVC from different systems and their pathological processes. Additionally, mechanisms of various stem cells through exosomes for the treatment of DVC were also discussed. For example, stem cells can secrete a variety of factors through the exosome pathway to promote the formation of new blood vessels. Furthermore, bone marrow mesenchymal stem cell-derived exosomes could inhibit the mTOR signaling pathway to enhance autophagy, which has a protective effect on kidney injury induced by hyperglycemia. In another article, Sun et al. described the multiple roles of exosomes in the pathology and treatment of diabetes and diabetic complications and emphasized that the pathological molecular spectrum formed by miRNAs and microproteins carried by exosomes from the body fluids of diabetic patients may provide possible new therapeutic ideas for DVC in the future.

Regarding the critical role of adipose tissue-derived exosomes in metabolic diseases such as destroying epithelial cells, disrupting blood vessels, aggravating liver fibrosis, and promoting the transformation of monocyte to both macrophage (M1 pro-inflammatory phenotype) and macrophage (M2 anti-inflammatory phenotype). Mei et al. reviewed recent advances in the role of adipose tissue-derived exosomes in metabolic diseases. In addition, current barriers hindering exosome-based therapeutic strategies are discussed.

Wang et al. discussed how organokines (adipokines, gut microbiota and their metabolites, intestinal cytokines, myokines, and hepatokines) and exosomes (adipocytes, skeletal muscle, and hepatocyte-derived exosomes) act as important triggers for adipose tissue macrophage recruitment and adipose tissue macrophage polarization, providing further insight into obesity treatment. In addition, they highlighted the complex interactions between various organokines and exosomes in the obese state, further revealing new pathways for the recruitment and polarization of adipose tissue macrophages.

The liver plays a vital role in modulating energy homeostasis. In modulating systemic glucolipid metabolism, most of the attributions for the release of hepatokines that maintain metabolic homeostasis in autocrine, endocrine and paracrine pathways that govern connections between the liver and other organs. Chen et al. (11) highlighted the interaction of some feeding-induced hepatokines such as Adropin, MANF, Leap2 and PCSK9 in the liver and extrahepatic tissues such as brain, adipose, heart, and pancreatic tissues, and also clarified the potential mechanisms by which these hepatokines mediate crosstalk between the liver and other organs. Targeting these feeding-induced hepatokines is expected to be a possible therapeutic approach for T2DM to help in control and treatment.

In conclusion, exosomes and organokines exert immunomodulatory functions through a variety of novel mechanisms that are valuable for the prediction and treatment of diabetes and its complications, obesity, and some metabolic diseases. The original research articles and review articles included in this issue present a range of topics that are under active investigation. With extensive investigations, additional pathological/beneficial roles of exosomes and organokines in diabetes and its complications and metabolic diseases are revealed to improve our understanding of exosomes and organokines and to elucidate the development of exosomal and organokines-based therapeutic strategies for more accurate diagnosis and more effective treatment of metabolic diseases. However, despite the recognition of the importance of exosomes and organokines in metabolic diseases, the specific regulatory mechanisms of these diseases are not fully understood and should remain a key focus of future research.

## Author contributions

X-HX, Q-YW, JL and Z-ZL conceived and wrote the manuscript. All authors contributed to the article and approved the submitted version.
